# Histopathological and Molecular Evaluation of the Experimentally Infected Goats by the Larval Forms of *Taenia multiceps*

**Published:** 2019

**Authors:** Omidreza AMRABADI, Ahmad ORYAN, Mohammad MOAZENI, Hassan SHARI-FIYAZDI, Maryam AKBARI

**Affiliations:** 1. Department of Pathology, School of Veterinary Medicine, Shiraz University, Shiraz, Iran; 2. Department of Parasitology, School of Veterinary Medicine, Shiraz University, Shiraz, Iran; 3. Department of Clinical Sciences, School of Veterinary Medicine, Shiraz University, Shiraz, Iran

**Keywords:** Coenurosis, *Coenurus cerebralis*, DNA (*CO1* and *ND1*), *Taenia gaigeri*, *Taenia multiceps*

## Abstract

**Background:**

Introduction of *Taenia multiceps* and *T. gaigeri* as two separate species have been recognized mainly on morphological grounds. This experimental study was conducted to test whether cerebral and non-cerebral forms of *Coenurus cerebralis* belong to one origin or they are originated from two different tape worms.

**Methods:**

Two groups of dogs were infected with the cerebral and muscular sources of the coenuri cysts. About two months later the eggs were collected from the fecal samples to be used to experimentally infect other healthy goats. Histopathological and molecular evaluation was conducted in two groups of goats that were challenged with *T. multiceps* eggs obtained from the infected dogs by brain and muscular sources of coenuri cysts in School of Veterinary Medicine of Shiraz University, Shiraz, Iran in 2015. All aberrant sites of predilection of the metacestode in goats were muscles, heart, diaphragm and lungs. The brain and spinal cord were carefully dissected and examined but the cysts were not found in these locations. In addition, the molecular genetic markers of mitochondrial DNA (*CO1* and *ND1*) were applied to resolve the questionable relationship between *T. multiceps* and *T. gaigeri*.

**Results:**

The larval stages of *T. multiceps* in brain and in other aberrant sites, which showed similar morphological criteria, were monophyletic species.

**Conclusion:**

Therefore, *T. gaigeri* must be considered taxonomically invalid.

## Introduction

Tapeworms of the genus *Taenia* include parasites of mammals parasitizing carnivores as definitive hosts, and herbivores (or omnivores) as intermediate hosts. Neurological disorders and the central nervous system's involvement are common in most species below the human and simian primates (1–3). The metacestode stage of *T. multiceps* (*Coenurus cerebralis*), also called gid, sturdy or stagger, develops in the CNS of ruminants and its adult form is found in the intestine of dogs, foxes, coyotes, and jackals (4, 5). Coenurosis poses serious problems for sheep production (6, 7). It is worldwide in distribution but is more common in the developing countries of Africa and Asia (8). While the cysts most commonly develop in central nervous system of sheep the metacestode may reach maturity and the cysts develop in other organs such as heart and lungs as well as sub-cutaneous, intramuscular and peritoneal areas of goats referred as *T. gaigeri* (9). The occurrence of *T. gaigeri* in goats has been reported by different investigators including, in Bangladesh (10), in India (4, 11, 12) and in Iran (13, 14). Several reports of human coenurosis can be found in the literature (15–18).

The cysts often persist throughout the life span of the host (4), however, such evidence should be confirmed after developing an experimental animal model. Establishment of the cyst(s) in noncerebral tissues implies that they may be originated from another strain or genetic variant of the parasite or may indicate that the goats reflect a different response to *T. multiceps* (19–21). This issue is, of course, complicated because the effect of morphological and physiological variations on taxonomy of tapeworms is not clearly defined. In addition, enough information is not available regarding the degree of influence of the host species on morphology and physiology of tapeworms (22). Therefore, it is necessary to give an accurate description of the larval forms of this cestode.

The present study was carried out to elucidate the histopathological and molecular evaluation of the larval forms of *T. multiceps*. This was done in a series of experiments in which the eggs of *T. multiceps* with two different sources (brain and muscle cysts) were transferred by ingestion to goats.

## Materials and Methods

### Experimental animals

#### Dogs

Four female dogs of mixed breed (two for each experiment) were selected for experimental infection with protoscoleces of *T. multiceps* collected from the brain (strain B) and muscle (strain M) sources to generate eggs for experimental infection in goats. The dogs were kept in separate kennels in hospital of School of Veterinary Medicine of Shiraz University, Shiraz, Iran in 2015. Dog fed commercial dog food, and supplied with clean water *ad libitum*. They were dewormed one month before the experimental infection. The health of the dogs was routinely monitored throughout the period of the study. *Taenia multiceps* cysts collected from the naturally infected sheep (brain source: strain B) and goats (muscle source: strain M) from a local slaughterhouse were immediately processed after collection by removing tissues surrounding the cysts. This was followed by washing the cysts in phosphate buffer saline, pH 7.3, preserving them in a sterile recipient and maintaining them at 4 °C until time of infection. Each of the four months old dogs was orally infected with 350 protoscoleces (23), in such a way that two dogs were infected by brain cysts, another two dogs were infected by muscle cysts. Patency was evaluated by collecting fecal samples from the infected dogs once a week to detect taeniidae eggs, using the McMaster flotation technique (24). After four months, when the parasites had reached maturity, shown by presence of taenid eggs in the faeces, the dogs were humanly euthanized. The adult *T. multiceps* parasites (strain B and M) were removed from the small intestine and the gravid proglottids were separated to recover the eggs. After several washing in normal saline, the gravid proglottids were then teased in watch glasses and the eggs were collected and preserved at 4 °C before being subjected to the challenge process. All infective doses were made up from the same batch of eggs.

#### Goats

Twelve female kids used in this experiment were five months old, and fed commercial food and hay and water *ad libitum*. The animals were dewormed before starting the experiment. The adaptation period, from purchase to infection, was one month. Each goat was orally infected via a stomach tube.

The experimental design has been shown in Table 1. The goats in group A were infected with the eggs collected from the dogs infected with the muscular cysts (strain M) while the goats in group B were infected with the eggs obtained from the dogs fed with the brain cysts (strain B). Each of the 12 goats were similarly infected by 80,000–100,000 eggs of *T. multiceps*. The goats have euthanized four, eight and 12 months post infection (two animals in each group). After necropsy, the number and size of the cysts, as well as their location was recorded. The cysts were removed from the tissues, washed with phosphate buffer saline, pH 7.3, and used for DNA isolation. All tissues involved with these cysts were used for histopathological examination.

**Table 1:** Experimental infection of goats with the eggs of *Taenia multiceps*

***Groups***	***Eggs of T. Multiceps***	***N^a^***	***Case number***	***Euthanization time (months)***
A	Muscle source	2	1–2	4
		2	3–4	8
		2	5–6	12
B	Brain source	2	7–8	4
		2	9–10	8
		2	11–12	12

N^a^ number of animals in each group

### Molecular assay and sequence analysis

The genomic DNA from the cysts of brain and muscular sources in experimentally infected goats as well as the adult worms in dogs were extracted, using a commercially available kits (Qiagen DNAeasy; Qiagen, Valencia, California), according to the manufacturer’s protocol. In addition, one sample obtained from one degenerated larval stage in the liver of an infected goat was used in PCR assay.

The nucleotide sequences of the mitochondrial cytochrome *c* oxidase subunit 1 (*CO1*, 396 bp) and NADH dehydrogenase 1 (*ND1*, 462 pb) genes were amplified (25, 26). From the mitochondrial genome, part of the *CO1* was amplified with JB3/JB4.5 primers (5′-TTTTTTGGGCATCCTGAGGTTTAT-3′/5′-AAAGAAAGAACATAATGAAAATG-3′) and another from the *ND1* was amplified with JB11/JB12 primers (5′- AGATTCGTAAGGGGCCTAATA-3′/5′-ACCACTAACTAATTCACTTTC-3′) (14, 27, 28). Amplification was performed using the lyophilized PCR microtubes (Model Accu-power PCR PreMix; BioNeer Co., Seoul, Korea) and under the following cycling conditions: 94 °C, 30 sec (denaturation), 55 °C, 30 sec (annealing), 72 °C, 30 sec (extension) for 35 cycles, and followed by 72 °C for 5 min (final extension). For each set of PCRs, the negative (no-DNA and DNA from healthy cases) and positive controls were included. Prior to further molecular analyses, the quality and intensity of individual aliquots (5 μl) from the amplicons were verified following electrophoresis in ethidium bromide-stained 1.5%–2% agarose-TAE gels, under ultraviolet trans-illumination.

The products were directly sequenced, using a capillary DNA analyzer (ABI 3730; Applied Biosystems, Foster City, California), after sequencing the reactions with a Big Dye Terminator v. 3.1 Cycle Sequencing Kit (Applied Biosystems). The forward and reverse nucleic acid sequence data were used to construct a continuous sequence of inserted DNA. Further comparison of the continuous sequences was made with the previously available taeniids sequences of the mitochondrial DNA (*CO1* and *ND1*) in NCBI (National Center for Biotechnology Information), using CLCMainWorkbench5 software (CLCBio, Aarhus, Denmark).

### Pathology

The cysts and tissue specimens in vicinity of them were collected from the infected organs of goats and were fixed in 10% neutral buffered formalin. Following a routine histological tissue-processing procedure, the samples were embedded in paraffin blocks and serial sections were cut at a thickness of 4–5 μm and stained with hematoxyline and eosin (H and E).

### Animal ethics

This experiment was performed under the approval of the University Committee on Animal Ethics. Moreover, the recommendations of European Council Directive (86/609/EC) of Nov 24, 1986, regarding the protection of animals used for experimental purposes was considered.

## Results

### Dogs

**Fig. 1:**
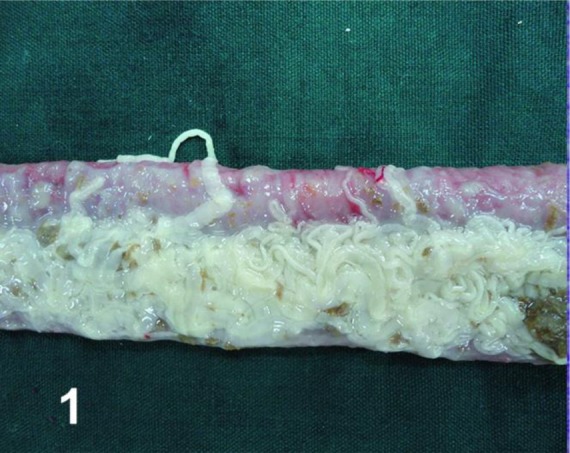
Jejunum of a dog heavily infected with large number of cestodes after four months post infection

**Fig. 2:**
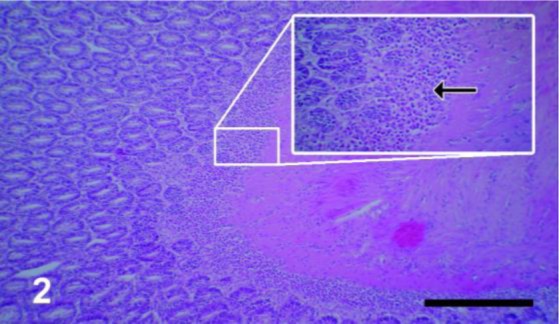
The submucosa of jejunum shows prominent cellular infiltration mainly consisting of numerous eosinophils after 56 d post infection. (HE, scale bar = 85μm). Inset: large numbers of eosinophils are seen (arrow) (× 400)

The first fully developed eggs shedding started 56 and 58 d after infection with the cysts of brain and muscle sources respectively, but all the four infected dogs were euthanized four months after feeding the cysts to achieve more segments containing fully-formed eggs. The small intestine, mainly the jejunum of all dogs, was packed and infested with large number of adult worms (Fig. 1). The average length of the worms was 60 cm with maximum of one meter. The jejunum was considerably thickened on palpation but was normal in appearance. The histopathologic examination revealed that the villi and lamina propria of the jejunum exhibited prominent cellular infiltration consisted of eosinophils, lymphocytes, plasma cells, and macrophages. The submucosa was the most severely affected layer and was permeated with numerous eosinophils, lymphocytes, plasma cells, and macrophages; therefore, this layer was greatly thickened (Fig. 2). No histopathological difference was seen in the infected dogs.

### Goats

#### Group A

**Fig. 3:**
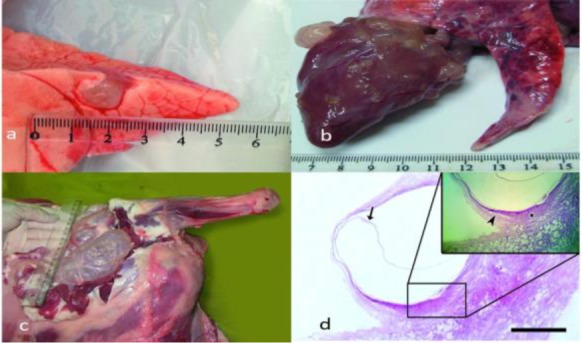
a) Apical lobe of the lung with a coenurus cyst in an experimentally infected goat infected by the eggs of muscle source *T. multiceps* four months post infection. b) Several coenuri cysts have invaded the heart of an experimentally infected goat infected by the eggs of muscle source *T. multiceps* four months post infection. c) Three coenuri cysts are embedded between the muscles of right thigh of an experimentally infected goat infected by the eggs of muscle source *T. multiceps* 12 months post infection. d) Section from the coenurus cyst in the lung four months post infection showing the metacestode wall (arrow) (HE, scale bar = 670 μm). Inset: infiltration of lymphocytes and proliferation of fibroblasts (asterisk) together with atelectasis of the alveoli and air passages is seen (arrowhead) (×40)

Five goats showed development of metacestodal cysts in their lungs, heart, diaphragm and muscles (Fig. 3 (a), (b) and (c)) at four, eight and 12 months post infection. However, one goat of this group showed no apparent infection after eight months post infection and after 12 months post infection in one goat there was a calcified lesion approximately 0.5 cm in diameter, on the anterior surface of the liver due to oncosphere migration based on molecular assay.

**Fig. 4:**
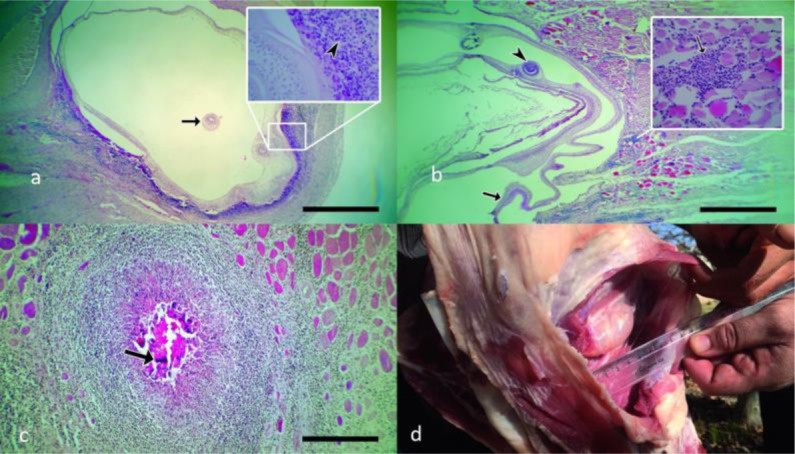
a) Photomicrograph of cardiac tissue of an infected goat showing early developing coenurus cyst with its premature protoscoleces (arrow) four months post infection. The cyst is surrounded by mononuclear cell infiltration (HE, scale bar = 210 μm). Inset: infiltration of lymphocytes, macrophages and eosinophils are seen (arrowhead) (×400). b) The arrowhead shows sucker of the larvae within the coenuri cyst four months post infection. The cyst has a thin wall (large arrow) and is surrounded by mononuclear cell infiltration (HE, scale bar = 210 μm). Inset: infiltration of lymphocytes is seen (small arrow) (×400). c) Granulomatus myositis due to coenurus larvae of an experimentally infected goat infected by the eggs of muscle source *T. multiceps*, 12 months post infection, with central caseous necrosis surrounded by eosinophils, lymphocytes, epitheloid macrophages and giant cells (arrow) (HE, scale bar = 21 μm). d) A coenurus cyst embedded between the intercostal muscles of a goat experimentally infected by the eggs of brain source *T. multiceps* four months post infection

No cyst was found in the CNS of the infected goats. The cysts were mostly embedded in the connective tissues of the muscles of both thighs and at their posterior margin, below the iliacus and near the popliteal lymph nodes. Apart from the thigh, some cysts were deeply embedded among the muscle external to the humerus; others were developed between the right psoas major and the Sartorius muscles of the abdomen. All the above cysts were provided with a well-developed fibrous connective tissue capsule. The Histopathological examination revealed infiltration of lymphocytes, plasma cells, macrophages and eosinophils together with proliferation of fibroblasts and fibrous connective formation around the cysts in the infected tissues. The infected lungs were atelectatic and the heart demonstrated chronic myocarditis together with fibroblasts and fibrous connective tissue proliferation (Figs. 3(d) and 4(a)). Some of the infected muscles showed severe myositis and were severely infiltrated by lymphocytes, plasma cells, eosinophils and macrophages (Fig. 4(b)). Granulomatous myositis was observed in one goat euthanized at 12 months post infection. The granuloma was surrounded by inflammatory cells including eosinophils, lymphocytes, epithelioid macrophages and giant cells (Fig. 4(c)).

#### Group B

Three goats showed development of coenurus cysts in the muscles and lungs. In the infected goat euthanized four months after infection one cyst was embedded in the connective tissues of the muscles on the left side of the neck below the nuchal ligament between the brachiocephalic and semispinalis capitis muscles. A cyst of about seven cm in diameter was found on the left side of the thorax in the goat euthanized four months post infection too. This cyst was deeply embedded in the intercostal muscles between the last two ribs (Fig. 4(d)). The last positive case euthanized after eight months post infection showed only two cysts in the lungs. However, one goat of this group showed no apparent infection after eight months post infection. All the above cysts were provided with a well-developed fibrous connective tissue capsule. The brain and spinal cord of all the six goats were carefully dissected but no cysts were found in these locations. Two other goats showed no apparent infection at all after 12 months post infection. Number and distribution pattern of coenuri in the two groups of goats experimentally infected with brain and muscle sources of *T. multiceps* eggs are summarized in Table 2.

**Table 2:** Number and distribution pattern of coenuri cyst collected from the goats experimentally infected with the eggs of the cerebral and muscular sources of *Taenia multiceps*

***Group***	***Source of infection***	***Case no.***	***Number of cysts/Location***
A	Muscle source	1	3/Forelimb muscles
		2	1/Lung
			14/Heart
		3	14/Hindlimb muscles
			4/Forelimb muscles
			3/Thorax muscle
		4	0
		5	1/Liver
		6	1/Hindlimb muscles
			2/Diaphragm
B	Brain source	7	1/Neck muscle
		8	1/Intercostal muscle
		9	2/Lungs
		10	0
		11	0
		12	0

### Molecular characterization

**Fig. 5:**
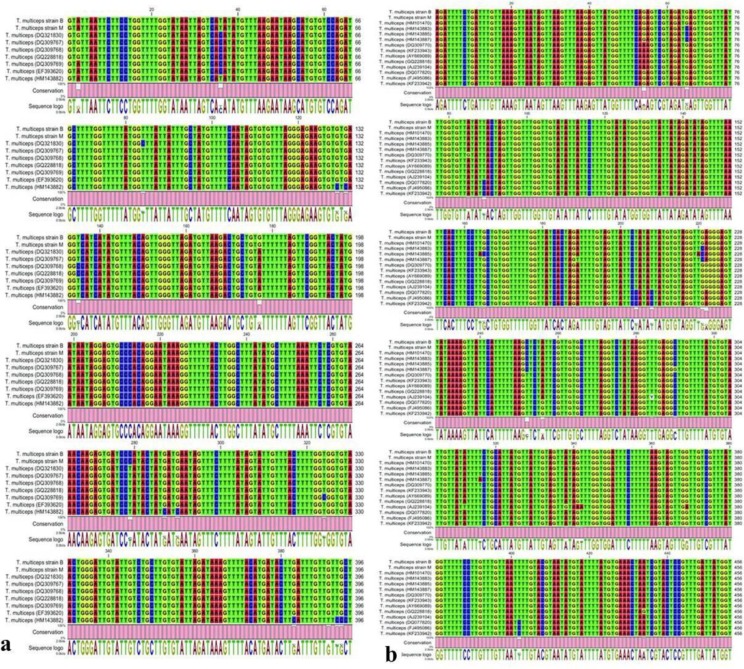
a) Partial Alignment of the *CO1* sequences for *T. multiceps* strains B and M compared to other strains existing in GenBank. b) Partial Alignment of the *ND1* sequences for *T. multiceps* strains B and M compared to other strains existing in GenBank

PCR was positive for all the examined samples (i, e. both sources of brain and muscular cysts, adult worms and all observed cysts (n= 46) in experimentally infected goats) by producing the expected fragments for *CO1* and *ND1* genes. Sequence analysis showed that all samples examined in our study were 100% identical to each other based on both mitochondrial markers. However, intraspecific variations were detected between Iranian *T. multiceps* strains and other strains existing in GenBank database based on *CO1* (0–3.03%) and *ND1* (0–2.85%). Pairwise comparison of Iranian *CO1* and *ND1* sequences are presented in Fig. 5 (a) and (b). The GenBank accession numbers were provided for *CO1* (KP663642 and KP663641) and *NDI* (KP663640 and KP663639) for muscular cysts developed in experimentally infected goat with the eggs of *T. multiceps* strain B and strain M, respectively.

## Discussion

In most infected cases, the cysts locate, grow, develop and create focal lesions with no clinical signs in non cerebral organs (4). Our findings throughout the experimental examination gave an accurate description of the larval forms of this cestode. Most of the goats were successfully infected by *T. multiceps* eggs of both muscle and brain origins. The infected goats, whether of the brain sources or musculature origins, had shown coenuri cysts in musculoskeletal muscles, heart, diaphragm, and lungs. No cyst was developed in the CNS of goats even after 100000 dose inoculation of the eggs. Palpable swellings with uneasiness, poor walking ability, poor appetite, and general discomfort were seen in the thigh muscles of the infected goats. Most of the cysts felt firm rather than fluctuant and exceeded 7 cm in diameter. All the cysts in goats were provided with well-developed adventitious capsules, consisted of fibrous connective tissue, newly formed blood vessels, fibroblasts and fibrocytes.

Although the common predilection sites of *T. multiceps* cysts in sheep is normally the CNS, the cysts in the goats of the present study, mainly developed in the intermuscular fascia, lungs, diaphragm and myocardium. The degenerating larval, proved by PCR, was found in the liver. Whether death of the larvae at this stage is attributable to the damage caused by the earlier complement and cellular attack, or to subsequent immunological attack is unknown but may involve a combination of these two mechanisms. Lack of infection in some of the goats euthanized after 12 months post infection in group B may be related to the age of the cysts and the cysts did not persist for a long time in the infected organs of the goats. Therefore, the cysts had been degenerated, disintegrated and disappeared earlier than this stage. Therefore, the difference in habitat of the larval stage of this metacestode appears to be related to the species of the host and not to the parasite.

This is probably due to the host differences and seems to be governed by factors such as the quantum and periodicity of infection in-take, subsequent *in situ* ongoing events of the host-parasite interaction and the acquired immune status of the goats.

A general description of the two species, *T. multiceps* and *T. gaigeri* (6) have been based on their morphology. Therefore, identification of *T. multiceps* and *T. gaigeri* into two separate species, based on vague morphological characters (29) is not convincing. Species of the genus *Taenia* are not easily identifiable by morphological means since many of the characters overlap (30). The extra-cerebral cysts in goats were due to *C. gaigeri* (31). The occurrence of non-cerebral coenurosis in goats may indicate that *T. gaigeri* is really a valid species (20) or at least a different strain of *T. multiceps* similar to that proposed (32) for *T. skrjabini* in Kazakhstan. No significant differences were found in morphological features between the *T. multiceps* and *T. gaigeri* metacestodes (19) and morphological identification of *T. gaigeri* from *T. multiceps* is not feasible (19). Recently, *T. gaigeri* may be synonymous with *T. multiceps* and *C. cerebralis* and *C. gaigeri* has been considered to be a non-analogous metacestode for the same tapeworm (21).

For effective diagnosis, treatment and control of parasitic diseases, parasite isolates should be readily and reliably identified (33). Molecular assay was employed, in the present study, to elucidate the similarities and differences of the gene sequences of coenuri cysts of muscular and cerebral sources in goats. The previous studies demonstrated the use of *CO1* and *ND1* genes of larval stage of *T. multiceps* from brain and muscle sources for molecular characterization (14, 34–36). Therefore, this molecular genetic study was conducted to characterize the DNA sequence of the *T. multiceps* metacestode and compare it with other accessible taeniid data from other parts of the world. The results of the present study showed that all samples either from the natural or experimental infection, brain or muscular sources, in the present study were 100% identical to each other at *CO1* and *ND1*. Similarly, comparative sequence analyses did not indicate the existence of genetically different host-adapted variants for this parasite in sheep and goat (36).

Pairwise comparison between the *CO1* and *ND1* sequences of our samples and other *T. multiceps* genotypes existing in GenBank showed a noticeable genetic variability, especially in *CO1* gene.

## Conclusion

The former view which presented *C. gaigeri* as a new strain is supportable only by evidence of marked host specificity. Our interpretation appears to be largely the result of placing emphasis on host (goat) induced variation in larval cestode developing in other than their natural host (sheep). Therefore, the main aim of the present study was to clarify the latter taxon (*gaigeri*) must be considered invalid. This metacestode does not develop in all the infected goats. More molecular markers and immunological studies are needed to clarify the host-parasite interaction.
